# Incommensurable Worldviews? Is Public Use of Complementary and Alternative Medicines Incompatible with Support for Science and Conventional Medicine?

**DOI:** 10.1371/journal.pone.0053174

**Published:** 2013-01-30

**Authors:** Paul Stoneman, Patrick Sturgis, Nick Allum, Elissa Sibley

**Affiliations:** 1 Department of Sociology, University of Surrey, Guildford, Surrey, United Kingdom; 2 National Centre for Research Methods, University of Southampton, Southampton, United Kingdom; 3 Department of Sociology, University of Essex, Colchester, United Kingdom; Children’s Hospital of Eastern Ontario, Canada

## Abstract

Proponents of controversial Complementary and Alternative Medicines, such as homeopathy, argue that these treatments can be used with great effect in addition to, and sometimes instead of, ‘conventional’ medicine. In doing so, they accept the idea that the scientific approach to the evaluation of treatment does not undermine use of and support for some of the more controversial CAM treatments. For those adhering to the scientific canon, however, such efficacy claims lack the requisite evidential basis from randomised controlled trials. It is not clear, however, whether such opposition characterises the views of the general public. In this paper we use data from the 2009 Wellcome Monitor survey to investigate public use of and beliefs about the efficacy of a prominent and controversial CAM within the United Kingdom, homeopathy. We proceed by using Latent Class Analysis to assess whether it is possible to identify a sub-group of the population who are at ease in combining support for science and conventional medicine with use of CAM treatments, and belief in the efficacy of homeopathy. Our results suggest that over 40% of the British public maintain positive evaluations of both homeopathy and conventional medicine simultaneously. Explanatory analyses reveal that simultaneous support for a controversial CAM treatment and conventional medicine is, in part, explained by a lack of scientific knowledge as well as concerns about the regulation of medical research.

## Introduction

For some, Complementary and Alternative Medicines (CAM), such as reiki, acupuncture, herbal medicines, homeopathy, and healing crystals offer a ‘natural’ and effective alternative to conventional medicine, which is overly-dependent on the synthetic remedies of multinational ‘big pharma’. Proponents of CAM argue that the treatments they espouse can be used with great effect alongside, and even instead of, conventional medicine [1] and that those who oppose complementary approaches are wedded to a narrow and restrictive view of both medical practice and treatment evaluation [2]–[5]. CAM treatments, moreover, cannot easily be consigned by their critics to the realm of snake-oil and quackery. For, despite often being questioned on the grounds of lacking robust evidence of clinical efficacy, they are nonetheless routinely prescribed in modern healthcare systems around the world, including those that are publically funded, such as the National Health Service in the United Kingdom [6]. Furthermore, some therapies commonly categorised as CAMs, such as massage, osteopathy and chiropractic care, have been evaluated with the rigorous methods of randomised controlled trials (RCTs) and have been shown to be safe and efficacious [7]–[9].

There are other forms of CAM, however, that have been the subject of intense scrutiny and critique from sections of the scientific community [10]–[11]. Perhaps because of the seemingly widespread acceptance of the merits of CAM within the general public and amongst many medical practitioners, these more controversial treatments have faced sustained opposition from those who advocate an evidence-based approach. Homeopathy, in particular, has been the source of sustained criticism from scientists on both evidential [12]–[16] and plausibility grounds [6], [17]–[19]. Such concerns relate not only to the quality and robustness of the underlying science but also to the consequences of patients relying on demonstrably inefficacious treatments when conventional medicines have, or should have been prescribed, with potentially fatal consequences [20]. This and other critical evidence led a recent UK Parliamentary Select Committee to recommend that homeopathy should not be funded through the National Health Service and that all regulatory licenses allowing homeopathic products to be sold as medicines should be withdrawn [6].

From the (what we shall call) ‘strong scientific’ perspective, then, there is little or no evidence to support the contention that homeopathy can be a useful and safe complement, let alone alternative to, conventional treatment [21]. For those adhering to this strong scientific position, support for the principles, processes and structures of conventional medicine must be considered *fundamentally incompatible* with a belief in the validity of treatments which have no evidence of clinical efficacy nor a plausible underlying mechanism [22]. Yet, while binary opposition between support for controversial CAM treatments such as homeopathy and conventional medicine is how the positions are often characterised within prominent public and scientific discourse [20], [23], it is not clear whether this is an accurate characterisation of the beliefs and behaviours of the general public. Do citizens adhere to a ‘science versus CAM’ binary opposition, or do they (at least in part) feel comfortable in supporting conventional *and* scientifically controversial alternative treatments simultaneously? If so, how can this apparent inconsistency be accounted for?

To address these questions, we use recent survey data from Britain to determine whether it is possible to identify a sub-group of the general population who maintain positive evaluations about *both* conventional medicine and homeopathy. To do this, we fit latent class models to a range of attitudinal indicators in order to assess whether a distinct group with ostensibly ‘incommensurable’ perspectives on treatment efficacy can be identified. We then use multinomial logistic regression to test which of a range of factors are predictive of membership of this sub-group. We conclude with a consideration of the implications of our findings for understanding the use of complementary and alternative medicines within the general public.

## Data and Methods

The data for our analysis are drawn from the 2009 Wellcome Trust Monitor survey of public knowledge, interest and engagement in biomedical science. The Wellcome Monitor uses a stratified, multi-stage probability sample design, with the Postcode Address File (PAF) used as the sampling frame of households. One adult member, aged 18 or above, of each responding household was randomly selected for interview using the Kish grid procedure [24]. The survey achieved a response rate of 49% using AAPOR Response Rate [25], yielding 1,179 adults as our analytical sample size (see [26] for full technical details of the survey). The strength of the Monitor for our purposes here is that in addition to asking about their use of a number of different CAM treatments, it also asks respondents to report their reasons for taking (and not taking), homeopathy. Respondents are also asked to assess how effective they think homeopathy is relative to conventional medicine. In order to classify the population in terms of their use of and beliefs about alternative and conventional medicine, we combine these CAM items with five additional questions. The first gauge attitudes towards science and conventional medicine. While no direct indicators of belief in science and conventional medicine are available in the Monitor survey, there is one item which asks about the importance of science in education which will be used alongside measures of trust in conventional medical practitioners and optimism about the potential of genetic science to produce medical advances in the future. Together, these tap into three important facets of support for science and conventional medicine: a belief in the importance of science in the core educational curriculum of all young people, faith in science’s ability to improve human health and longevity through technological innovation, and a positive orientation toward the primary ‘face’ and first port of call within the conventional medical system. Due to the large number of cells produced by the cross-classification of these five variables, all indicators are recoded into binary format in order to avoid estimation problems due to sparse cell sizes. Full question wordings and details of the variable coding are presented in Form S1.

### Methods

As our primary objective in the multivariate analyses is to determine whether it is possible to identify a sub-group of the population who appear to simultaneously hold favourable attitudes toward both science and conventional medicine *and* CAM, an appropriate methodology is Latent Class Analysis (LCA). LCA can be thought of as conceptually equivalent to Factor Analysis, but for manifest and latent variables which are categorical, rather than continuous [27]. The key underlying rationale of LCA is that the observed associations between the manifest variables can be explained by the K-class latent variable, which is to say that the manifest variables are conditionally independent, controlling for the latent variable [27].

The first question that must be addressed in conducting a LCA is how many categories, K (k = 1…K), there should be in the categorical latent variable. Typically, analysts fit models with an increasing number of latent class categories and then select the model with the best fit to the data. Because the difference in the likelihood ratio cannot be used to select between models with different numbers of latent classes [28], optimal fit is determined by information-based measures such as Akaike’s Information Criterion (AIC) and the Bayesian Information Criterion (BIC), with the lowest value on these measures taken as indicating the best fit to the data [29]. Once the optimal number of categories in the latent class variable has been determined, interpretation of the derived latent categories is undertaken. As with factor analysis, this is an essentially inductive process, with the meaning of the latent class groups derived from the pattern of association between the latent classes and the manifest variables. For instance, one can examine the estimated posterior probabilities of response to the manifest variables, for each of the different latent classes [30].

In addition to examining how different sub-groups within the general public orient themselves toward conventional and alternative medicine, we also wish to understand the factors which characterise membership of the different latent class groups that we observe. Previous analyses have revealed some robust patterns relating to demographic characteristics, with CAM users consistently found to be younger [31]–[32] (31 Braun et al. 2010; Hyland, Lewith & Westoby 2003), female [33]–[36], better educated [33], [37]–[39] and suffering from poor health and chronic illness [34], [37], [40]–[43]. In line with these analyses, we will use multinomial logistic regression to assess the extent to which different orientations toward conventional and alternative medicine are a function of such variables. We will also test the effects of two more contextualised variables relating to individual orientations towards science and conventional medicine, namely three different measures of science knowledge – factual scientific knowledge, understanding of probability, and understanding of the process of science [44] – as well as four indicators of positive and negative concerns expressed by respondents about medical research. Item wordings and descriptive statistics for all three knowledge items plus the medical concern variable are provided in Form S2. Models are estimated using MPlus 6.1 [45] and the coding for all variables included in the multinomial regression are provided in Form S3.

## Results

**Figure 1:**
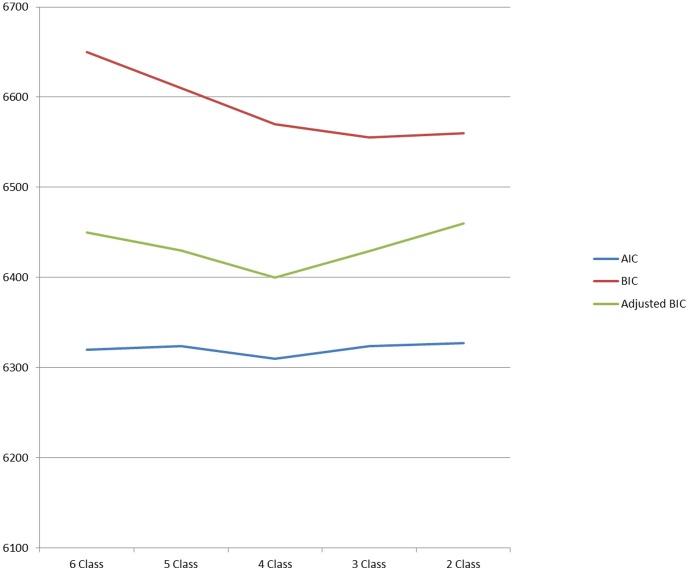
AIC and BIC values for latent class models.

Figure 1 plots the BIC, adjusted BIC and AIC values for latent class models with an incrementally decreasing number of classes. Because the model with the lowest value on these criteria should be considered the best fit to the data, figure 1 indicates that the 4 class model should be preferred on empirical grounds alone, with the adjusted BIC and AIC having the lowest value for this model, although the unadjusted BIC shows a slight improvement in fit, moving from the 4 to the 3 class model. However, although the latent class model reached its minimum on two of the measures for the 4 class model, we encountered irresolvable convergence problems with the 4 class model, which meant that it was not possible to estimate standard errors for the model coefficients. Examination of the pattern of model coefficients additionally indicated that the fourth class was very similar to the third class in the three class model, representing what Muthen (2001) has termed a ‘splinter class’ [45]. A splinter class is a class which, while empirically distinguishable from a larger class, is nonetheless very similar in substantive terms and so may be dropped on the grounds of parsimony. For these reasons, then, we select the three class model as our preferred solution.

Table 1 shows, for the preferred 3 class model, the estimated posterior probabilities of response to each manifest variable. Class 1 represents just over a quarter of the total sample (27%) and comprises of individuals who have the lowest probability of being supportive of science in compulsory education, who are unlikely to believe homeopathy is effective, and who are unlikely to have used a CAM treatment. While class 1 is broadly trusting of doctors, they are less trusting than those in class 2, and are unlikely to be optimistic about the potential for advances in medicine based on genetic science, relative to the other two classes. For these reasons, we use the term ‘disaffected’ to refer to class 1, as their attitudes and reported behaviours reflect a general lack of endorsement of both CAM *and* science/conventional medicine. Class 2 makes up nearly a third of the total sample (32%) and contains individuals who are strongly supportive of science being taught as a core part of compulsory education, have the highest probability of expressing trust in doctors and the highest probability of being optimistic about the likelihood of medical advances through genetic science. On the other hand, those in class 2 have a low probability of reporting CAM use and are even less likely to evaluate homeopathy as being effective, relative to conventional medicine. We therefore refer to latent class 2 as ‘conventional’, in the sense that they broadly reject the efficacy of homeopathy but are strongly supportive of and optimistic about conventional medicine.

**Table 1:** Estimated posterior probabilities from Latent Class Analysis.

	Latent class 1(n = 326)	Latent class 2(n = 375)	Latent class 3(n = 478)
*Is a science education ‘very important’?*			
No	0.47	0.08	0.15
Yes	0.53	0.92	0.85
*Is Homeopathy effective?*			
No	0.89	0.91	0.55
Yes	0.11	0.09	0.45
*Used CAM?*			
No	0.77	0.73	0.22
Yes	0.23	0.27	0.78
*Trust doctors?*			
No	0.27	0.20	0.37
Yes	0.73	0.80	0.63
*Optimistic medical advances?*			
No	0.48	0.01	0.09
Yes	0.52	0.99	0.91

Finally, latent class 3 represents the largest class (41%) and is the only class with a high probability of reporting having used CAM as well as rating homeopathy as effective, relative to conventional medicine. However, the positive orientation toward alternative medicine is not accompanied by a concomitant rejection of science and conventional medicine, for latent class 3 also has a high probability of expressing strong support for science in education, of trusting conventional medical practitioners, and of being optimistic about the likelihood of medical advances coming about through developments in modern genetic science. Thus, the pattern of responses observed for latent class 3 appear to confirm that, counter to the tenor of elite discourse, there is a sub-group of the population who are *simultaneously* supportive of both science and conventional medicine and CAM. Due to the apparently contradictory nature of the attitudes and behaviours which characterise this group, we refer to latent class 3 as the ‘dissonant’ class.

Having identified a population sub-group whose orientation toward both conventional and alternative medicine is positive as well as a ‘disaffected’ group, we now proceed to estimating predictive models of class membership in order to obtain a clearer understanding of the factors which give rise to such an outlook. Table 2 presents the results of the multinomial regression for the 3 class model. The reference category for the dependent variable is membership of the ‘conventional’ group, so the coefficients are interpreted as the (log of) the odds of being in the disaffected (column 1) or the dissonant (column 2) class, relative to the conventional class, for each unit change in the predictor. Thus, we can see that membership of the disaffected group is more likely amongst women, those who do not have a science qualification, are less interested in medical research and have lower levels of scientific knowledge. This is true for all three measures of scientific knowledge, although the coefficient for understanding the experimental method is not statistically significant. None of the attitudinal measures of concern about medical research differentiate the ‘disaffected’ from the ‘conventional’ latent class. For the dissonant class, the pattern is similar, although with some notable differences. Women are, again, more likely to be in this class than men, as are people who do not have a science qualification. However, interest in medical research and science does not distinguish between the dissonant and the conventional latent class groups and membership of the dissonant class is more likely amongst people with higher levels of educational qualification. The probability of membership in the dissonant class increases as scientific knowledge decreases, but only for the measure of factual scientific knowledge; understanding of probability and the experimental method do not differentiate these two classes. Of the attitudinal measures, only those expressing concerns relating to a lack of regulation of medical research are more likely to be in the dissonant class.

**Table 2:** Multinomial Logistic regression predicting latent class membership

*Reference class = Conventional*	*Disaffected*	*Dissonant*
	B (S.E.)	B (S.E)
Intercept	12.63 (1.20)	2.60 (1.50)
Age	-0.33 (.12)	-0.31 (.17)
Sex (male = 1)	**-1.17** (.43)	**-1.66** (.35)
Education (qualification level)	-0.036 (.12)	**0.23** (.10)
Has science qualification	**-0.69** (.35)	**-0.49** (.22)
Interest in science	-0.38 (.23)	-0.13 (.17)
Interest in medical research	**-1.38** (.35)	0.42 (.30)
Disability / Long term illness	0.17 (.23)	0.13 (.20)
Science knowledge		
*Factual*	**-0.75** (.16)	**-0.35** (.14)
*Method*	-0.29 (.40)	0.38 (.36)
*Probabilities*	**-0.82** (.40)	0.13 (.35)
Concerns about medical researc		
*Too little regulation*	0.22 (.28)	**0.45** (.23)
*Too slow*	0.29 (.22)	0.21 (.17)
*Risks and cost*	-0.20 (.28)	-0.33 (.21)
*Too much regulation*	0.03 (.87)	0.97 (.66)
N	1179	

Coefficients are logits; standard errors in parentheses; bold indicates that coefficients are statistically significant at the 95% level of confidence, or below.

In summary, then, we find some support for the idea that use of CAM is associated with a lack of scientific knowledge, although the effect is rather more nuanced than the theoretical literature might lead us to expect. Only one of the important domains of science knowledge is significantly related to membership of the dissonant latent class group and the predictive strength of science knowledge is actually higher for the latent class who reject both conventional *and* alternative medicine. The fact that having no science qualification is also significantly associated with membership of the dissonant class adds some weight to the inference that a positive orientation toward CAM, whether coupled with a rejection of conventional medicine or not, is somehow related to a lack of understanding of science. However, the fact that having higher qualifications is also diagnostic of membership of the dissonant group indicates that the effect is rather nuanced and is not reflective of a more general cognitive deficit.

## Discussion

The use of and claims made for a number of apparently inefficacious complementary and alternative medicines continues to evoke controversy and alarm amongst scientists and medical practitioners throughout the world [20], [46]. In particular, many have expressed frustration and concern that controversial CAM treatments such as homeopathy appear to be enjoying increasing popularity as scepticism about the producers and purveyors of conventional medicine grows [6]. Not only is the use of such treatments seen as a waste of scarce resources, particularly when funded through government expenditure, it also represents a threat to public health if citizens turn to inefficacious treatments in preference to conventional medicine. From this perspective then, support for conventional medicine and treatments such as homeopathy are seen as logically incompatible. One either follows the strictures of science and of evidence based medicine, or one does not. It would be, from this perspective, seemingly irrational to believe in the evaluation of treatment efficacy via RCTs while also using, or believing in the efficacy of treatments such as homeopathy. However, while the underlying issues as well as the positions of the competing camps in this debate are now well rehearsed, considerably less is known about how the general public orientate themselves toward these two dominant systems of health practice and belief. Our objective in this paper has been to investigate the attitudes and behaviours of the general public toward controversial CAM treatments in greater detail than has been evident to date. With a focus on the highly controversial case of homeopathy, we have sought to establish whether public attitudes can be characterised as falling into starkly oppositional ‘camps’, or whether citizens are, as in other domains, untroubled by combining apparently contradictory practices and beliefs.

Our analyses confirm the findings of existing survey evidence, that a large proportion of the general public have, at some time in their lives, made use of CAM treatments. It is clear that use of CAM is very widespread throughout the general population which, of course, reinforces the pertinence of arguments about its potentially malign effects on public health. From where, though, does use of and belief in the efficacy of CAM emanate? We found evidence that around a third of the UK public appear to espouse what we have termed a ‘conventional’ orientation, in that they express optimism about and trust in conventional medicine and science, while also rejecting the use of CAM in general and the efficacy of homeopathy in particular. However, the largest sub-group identified in our analysis was characterised by what we termed a ‘dissonant’ orientation; support for and optimism about conventional medicine and science alongside use of CAM and belief in the efficacy of homeopathy. These results raise questions about the validity of commonly advanced explanations for CAM use; that it is, in some simple manner, driven by anti-science attitudes and a rejection of conventional medicine [11], [47]. On the contrary, while this may be true of some CAM users, many are clearly quite comfortable in maintaining positive orientations toward both conventional and alternative forms of medicine.

In order to account for this heterogeneity in orientation toward use of and beliefs about CAM, a number of explanatory variables were included in a subsequent regression model to predict latent class membership. This revealed that the ‘dissonant’ sub-group had lower levels of scientific knowledge and was less likely to have a qualification in a scientific discipline. This is consistent with the idea, then, that the public’s positive evaluation of controversial CAM treatments such as homeopathy is, at least in part, due to a failure to properly understand the evidential basis of the conclusions drawn by the scientific community - that many of these treatments are not efficacious at all. Without a proper understanding of the principles of experimental design, of probability theory and of anonymous peer review, it is likely that the more anecdotal and selective evidence garnered in support of homeopathy, reiki, healing crystals and so on will be more persuasive. The regression models also showed that the ‘dissonant’ group’s positive evaluation of CAM is related to a perception that medical research is under-regulated. This suggests that some people turn to CAM treatments as a result of concerns about the adequacy of the protections that are in place to guard against improper or unsafe practice in the governance of conventional medicine.

As ever, the results of our analyses raise as many questions as they answer. We have shown that positive beliefs about CAM are connected to scientific knowledge but our interpretation of the mechanism is, at this stage, speculative. Additionally, our evidence is based largely on responses relating to only one type of CAM treatment, homeopathy. Albeit that this is one of the most popular and controversial CAM treatments currently available, we cannot be certain that our results will generalise to other less controversial CAM treatments, or indeed, to other social and cultural contexts. If CAMs such as homeopathy genuinely represent a threat to public health, rather than a benign matter of personal freedom, it is clear that we need to understand more about the factors which lead to and maintain beliefs about its utility. In this paper, we have taken important steps toward developing a framework for better understanding public orientations toward alternative medicine and, we hope, signalled some potentially fruitful avenues for future research.

## Supporting Information

Form S1. Coding for variables in latent class models. (DOCX)

Form S2. Coding for science knowledge and medical concern variables. (DOCX)

Form S3. Coding of dependent variables in multinomial linear regression. (DOCX)
