# Supplementary material for: Incommensurable Worldviews? Is Public Use of Complementary and Alternative Medicines Incompatible with Support for Science and Conventional Medicine?
Source: PLoS One. 2013 Jan 30;8(1):e53174. doi: 10.1371/journal.pone.0053174 (PMC3559728; doi:10.1371/journal.pone.0053174)
Supplement: Form S1. Coding for variables in latent class models. — (DOCX) [file pone.0053174.s001.docx]

**FORM S1: Coding for variables in latent class models**

**Importance of Science Education**

How important do you think it is that science is taught up to the age of 16 in school?

1. Very important

2. Quite important

3. Not very important

4. Not at all important

For this item, it is important to distinguish between those who think that science is an integral part of a primary, junior and secondary school education from those who think it is not a top concern. Answering ‘very important’ implies that a science education should be a top priority for a child’s education, while answering ‘quite important’ to this question implies that, while of *some* importance, science is not essential. As such, this variable is recoded into a binary variable where ‘very important’ is coded as ‘one’ (79%), and ‘quite important’, ‘not very important’ and ‘not at all important’ are coded as ‘zero’ (21%).

**Trust GPs/Nurses/Medical Practitioners**

Please tell me how much trust you have in each of the following to provide accurate and reliable information about medical research…Doctors, nurses and other medical practitioners? Please pick your answer from this card

1. Complete trust

2. A great deal of trust

3. Some trust

4. Very little trust

5. No trust at all

For this item, “complete” trust and “great deal” of trust are conceptually very close together being overtly positive, while “some trust” is a response tainted with caution. As such, in order to separate those who think of doctors/nurses/medical practitioners as mostly trustworthy from those who are more cautious, this variable will be recoded into a binary variable where ‘complete trust’ and ‘a great deal of trust’ are coded as ‘one’ (72%), and all other responses are coded as ‘zero’ (28%).

**Optimism About Medical Advance Based on Genetic Research**

Are you very optimistic about the possibility of medical advances as a result of genetic research, somewhat optimistic, not too optimistic or not at all optimistic?

1. Very optimistic

2. Somewhat optimistic

3. Not too optimistic

4. Not at all optimistic

With this item, “very optimistic” is obviously overtly positive and “somewhat optimistic”, while being a more reserved judgement, is also positive in nature. To separate those who are optimistic from those who are not, this variable will be recoded where the first two responses are coded as ‘one’ (84%), and the second two responses (‘not too optimistic’ and ‘not at all optimistic’) are coded as ‘zero’ (16%, includes “don’t know” answers).

**Lifetime Usage of CAM**

A binary variable is created where ‘one’ = respondent has recorded lifetime usage of at least one CAM (46%), ‘zero’ = respondent has recorded no lifetime usage of CAM (54%)

**Perceived Efficacy of Homeopathy**

People have different views about how effective homeopathy can be at treating illness. Compared with medical treatments available from your GP or other qualified medical staff, do you think that homeopathy can be…READ OUT….

1. …. More effective,

2. …. Just as effective,

3. …. Less effective than other medical treatments, or

4. …Not effective at all?

5 (SPONTANEOUS: Depends on the illness)

6 {SPONTANEOUS: Respondent doesn’t know what homeopathy is}

In order to separate those who think homeopathy is just as or more effective than conventional medicine from those who think otherwise, the first two positive categories are coded as 'one' (51%). This group compromising of just over half of the sample who know what homeopathy is also includes the spontaneous ‘depends on illness’ category as it demonstrates a positive attitude towards the effectiveness of homeopathy. The ‘less effective than other medical treatments’ and ‘not effective at all’ categories are coded as 'zero'. If the ‘respondent doesn’t know what homeopathy is’, then, by definition, the respondent cannot believe homeopathy to be effective relative to conventional medicine, so this category is also included in this final group coded as ‘zero’ (representing 49% of those who know what homeopathy is).
