# Supplementary material for: Incommensurable Worldviews? Is Public Use of Complementary and Alternative Medicines Incompatible with Support for Science and Conventional Medicine?
Source: PLoS One. 2013 Jan 30;8(1):e53174. doi: 10.1371/journal.pone.0053174 (PMC3559728; doi:10.1371/journal.pone.0053174)
Supplement: Form S2. Coding for science knowledge and medical concern variables. — (DOCX) [file pone.0053174.s002.docx]

**FORM S2: Coding for science knowledge and medical concern variables**

**Science Knowledge: Factual**

Now for a quick quiz about science. For each of the following statements, please say whether you think it is definitely true, probably true, probably false or definitely false. If you don't know, just say so and we’ll go on to the next one.

1. All plants and animals have DNA [TRUE] (89%)
2. The cloning of living things produces genetically identical copies [TRUE] (80%)
3. The oxygen we breathe comes from plants [TRUE] (80%)
4. By eating a genetically modified fruit, a person’s genes could also become modified [FALSE] (74%)
5. All radioactivity is man-made [FALSE] (69%)
6. It is the mother’s genes that determine the sex of the child [FALSE] (63%)
7. More than half of human genes are identical to those of mice [TRUE] (57%)
8. Lasers work by focussing sound waves [FALSE] (49%)
9. Electrons are smaller than atoms [TRUE] (43%)

Note: correct answers in parentheses, percentage of respondents choosing the right answer in brackets

**Science Knowledge: understanding of Scientific Process**

Suppose a drug used to treat high blood pressure is suspected of having no effect. On this card, there are 3 different ways scientists might use to investigate the problem. Which one do you think scientists would be likely to use?

1. Talk to those patients that have used the drug to get their opinion [FALSE] (16%)
2. Use their knowledge of medicine to decide how good the drug is [FALSE] (12%)
3. Give the drug to some patients, but not to others, then compare the results for each group [TRUE] (70%)
4. Don’t know (2%)

Note: correct answers in parentheses, percentage of respondents choosing the right answer in brackets

**Science Knowledge: Understanding Probability**

Now think about this situation. A doctor tells a couple that their genetic makeup means that they've got a one in four chance of having a child with an inherited disease…

1. Does this mean that if their first three children are healthy, the fourth will have the illness? [FALSE] (85%)
2. Does this mean that if their first child has the illness, the next three will not? [FALSE] (90%)
3. Does this mean that each of the couple's children will have the same risk of suffering from the illness? [TRUE] (77%)
4. Does this mean that if they have only three children, none will have the illness? [FALSE] (90%)

Note: correct answers in parentheses, percentage of respondents choosing the right answer in brackets

**Medical Concern**

Here is a list of concerns that people have raised about developments in medical research. Thinking about medical research, are you concerned about any of the things listed on this card?

1. Developments are taking place too fast [NEGATIVE] (6%)
2. Don’t know enough about future risks [NEGATIVE] (46%)
3. Too closely regulated [POSITI VE] (6%)
4. Too expensive [NEGATIVE] (21%)
5. Lack of rules to control what scientists can do [NEGATIVE] (18%)
6. Not enough attention paid to what public want [NEGATIVE] (24%)
7. Not enough money being spent on certain areas [POSITIVE] (55%)
8. Not progressing fast enough [POSITIVE] (21%)
9. Too much involvement from private companies [NEGATIVE] (25%)
10. Other (PLEASE SPECIFY)
11. None of these (9%)

Note: indication of positive/negative answer in parentheses, percentage of respondents choosing the answer in brackets

A LCA on these items revealed that they cluster around four different types of positive and negative responses:. (1) concern about a lack of regulation – that medical research moves “too fast”, there are a “lack of rules” to control what scientists do, and concerns are expressed about the “role of private companies” (negative); (2) concern about too much regulation (positive); (3) concerns that there are significant “future risks” and that it is “too expensive” (negative); (4) concerns that developments are “too slow” and there is too little money available (positive)
