# Supplementary material for: Incommensurable Worldviews? Is Public Use of Complementary and Alternative Medicines Incompatible with Support for Science and Conventional Medicine?
Source: PLoS One. 2013 Jan 30;8(1):e53174. doi: 10.1371/journal.pone.0053174 (PMC3559728; doi:10.1371/journal.pone.0053174)
Supplement: Form S3. Coding of dependent variables in multinomial linear regression. — (DOCX) [file pone.0053174.s003.docx]

**FORM S3: Coding of dependent variables in multinomial linear regression**

age = (1) 18-24; (2) 25-34; (3) 35-49; (4) 50-54; (5) 65+

sex = (0) female; (1) male

education = (1) no qualifications; (2) GCSEs; (3) 'A' levels; (4) degree; (5) postgraduate degree

science education = (1) none; (2) school; (3) college; (4) university

interest in science = (1) none at all; (2) some; (3) quite a lot; (4) a lot

interest in medical research = (1) none at all; (2) some; (3) quite a lot; (4) a lot

disability/long term illness = (0) no; (1) yes

science knowledge:

factual = ten-point scale ranging from 0 to 9 based on number of correct answers to quiz

method = binary variable where true answer is coded as 1, two false answers coded as 0

probabilities = binary variable where those who correctly state each of the four scenarios as true/false are coded as 1, all other combinations of answers are coded as 0

lack of regulation = total number of negative responses, four point scale

too slow = total number of positive responses, four point scale

risks = total number of negative responses, three point scale

too much regulation = (0) not mentioned; (1) mentioned
